# The Immune Response in Measles: Virus Control, Clearance and Protective Immunity

**DOI:** 10.3390/v8100282

**Published:** 2016-10-12

**Authors:** Diane E. Griffin

**Affiliations:** W. Harry Feinstone Department of Molecular Microbiology and Immunology, Johns Hopkins Bloomberg School of Public Health, Baltimore, MD 21205, USA; dgriffi6@jhu.edu; Tel.: 410-955-3459

**Keywords:** rash, viral RNA persistence, antibody maturation, inflammasome

## Abstract

Measles is an acute systemic viral infection with immune system interactions that play essential roles in multiple stages of infection and disease. Measles virus (MeV) infection does not induce type 1 interferons, but leads to production of cytokines and chemokines associated with nuclear factor kappa-light-chain-enhancer of activated B cells (NFκB) signaling and activation of the NACHT, LRR and PYD domains-containing protein (NLRP3) inflammasome. This restricted response allows extensive virus replication and spread during a clinically silent latent period of 10–14 days. The first appearance of the disease is a 2–3 day prodrome of fever, runny nose, cough, and conjunctivitis that is followed by a characteristic maculopapular rash that spreads from the face and trunk to the extremities. The rash is a manifestation of the MeV-specific type 1 CD4^+^ and CD8^+^ T cell adaptive immune response with lymphocyte infiltration into tissue sites of MeV replication and coincides with clearance of infectious virus. However, clearance of viral RNA from blood and tissues occurs over weeks to months after resolution of the rash and is associated with a period of immunosuppression. However, during viral RNA clearance, MeV-specific antibody also matures in type and avidity and T cell functions evolve from type 1 to type 2 and 17 responses that promote B cell development. Recovery is associated with sustained levels of neutralizing antibody and life-long protective immunity.

## 1. Introduction

Measles is a highly contagious systemic viral infection that remains one of the most important causes of worldwide morbidity and mortality in children despite the availability of a safe and effective live attenuated virus vaccine [1,2,3]. Recent strides have been made toward global measles control using a 2-dose vaccination approach, but logistical and financial difficulties in sustaining the current mass campaign strategy in developing countries have resulted in a resurgence in measles and measles deaths [4]. In addition, complacency and concerns about safety, along with philosophical and religious objections to vaccination, have resulted in measles being re-established as an endemic disease in many industrialized nations [5,6,7].

Measles virus (MeV), the causative agent of measles, is a human virus without an animal reservoir that is efficiently transmitted by aerosol or respiratory droplets. Although nonhuman primate populations are too small to sustain MeV transmission, study of macaques experimentally infected with wild type (WT) strains of MeV has provided much of our detailed knowledge of measles pathogenesis [8,9]. After introduction of MeV into the respiratory tract, immature pulmonary dendritic cells (DCs) or alveolar macrophages capture and transport MeV to regional lymph nodes (LNs) where the immune response is initiated, virus is amplified and spread of infection facilitated [10,11]. Infected immune cells (B cells, CD4^+^ and CD8^+^ memory T cells, monocytes) then enter the circulation and spread the virus to multiple lymphoid (e.g., spleen, thymus, LNs) and non-lymphoid (e.g., skin, conjunctivae, kidney, lung, liver) organs where it replicates in endothelial cells, epithelial cells, lymphocytes and macrophages [12,13,14,15,16,17,18]. 

**Figure 1:**
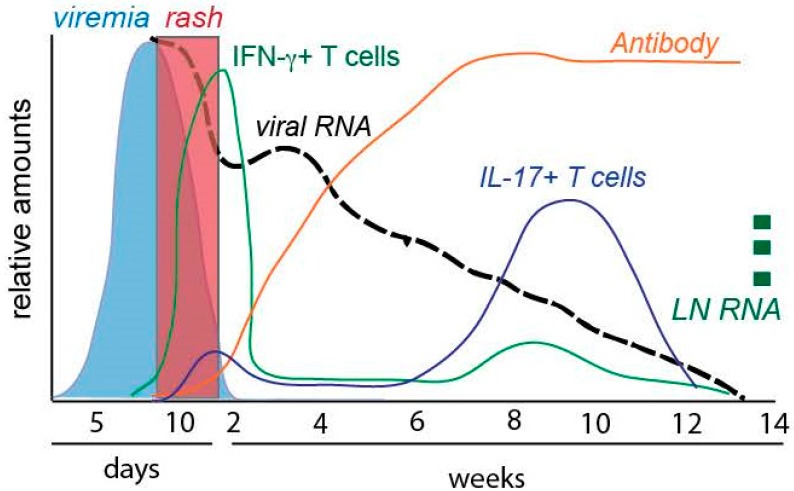
Schematic diagram of measles virus (MeV) clearance and immune responses in rhesus macaques. Infection with wild type (WT) MeV results in viremia (infectious virus) and rash. The rash is associated with appearance of interferon (IFN)-γ-producing T cells that decline quickly after the viremia is cleared. There is a prolonged phase of slow viral RNA clearance from peripheral blood mononuclear cells (PBMCs) with persistence of viral RNA in lymph nodes (LN). During RNA clearance antibody increases in amount and avidity. Waves of MeV-specific T cells continue to appear in circulation with a shift from IFN-γ production to interleukin 17 (IL-17) production. Data graphed from Lin et al. [9,20].

The immune response plays an essential role in multiple stages of infection and disease. The initial innate immune response is restricted due to inhibition of the interferon (IFN) response and allows extensive virus replication and spread during a clinically silent latent period of 10–14 days. The first appearance of the disease is a 2–3 day prodrome of fever, runny nose, cough, and conjunctivitis that is followed by the appearance of a characteristic maculopapular rash that spreads from the face and trunk to the extremities. The rash is a manifestation of the MeV-specific adaptive cellular immune response and coincides with clearance of infectious virus. However, clearance of viral RNA from blood and tissues is much slower than clearance of infectious virus and proceeds over weeks to months after resolution of the rash (Figure 1). The period of RNA persistence coincides with decreased host resistance to infection that can be prolonged [19]. Recovery is associated with life-long protection from MeV re-infection.

## 2. Innate Response

Typically, the innate response to RNA virus infection is dominated by infected cell production of types I and III IFNs. Induction of IFN occurs through recognition of viral RNA or protein by toll-like receptors or by cytoplasmic RNA helicases that lead to activation of the cytoplasmic transcription factors IFN regulatory factor (IRF)-3 and nuclear factor kappa-light-chain-enhancer of activated B cells (NFκB). Translocation of IRF-3 and NFκB to the nucleus induces transcription of the mRNAs for early response proteins such as Regulated on Activation, Normal T Cell Expressed and Secreted/Chemokine (C-C motif) ligand 5 (RANTES/CCL5), IRF-7 and IFN-β with subsequent induction of IFN-stimulated genes (ISGs) with antiviral activity including myxovirus resistance (Mx), adenosine deaminase acting on RNA 1 (ADAR1), ISG15, ISG56 and IFN-α that can act to suppress virus replication [21,22]. 

However, in the absence of defective interfering RNAs both induction of and signaling by IFN-α/β are effectively inhibited through the combined activities of the MeV P, C and V proteins [23,24,25,26,27,28,29,30,31,32] with little to no evidence of IFN induction during measles [32,33]. Therefore, IFN is not produced to suppress virus replication after infection resulting in a prolonged latent period during which there is systemic virus spread without signs or symptoms of infection. 

However, there is evidence for engagement of stress-response proteins and inflammasome activation by MeV infection. In antigen presenting cells (APCs) MeV interaction with DC-specific intercellular adhesion molecule-3 grabbing non-integrin (DC-SIGN) suppresses RNA helicase activation so that infection increases expression of stress-induced genes without inducing IFN [31,34]. In vitro studies show that MeV infection of myeloid cells stimulates assembly of the NACHT, LRR and PYD domains-containing protein (NLRP3) inflammasome in a mitofusin 2-dependent process with activation of caspase-1 followed by cleavage and secretion of mature interleukin (IL)-1β and IL-18 [35,36]. Transcriptional analysis of peripheral blood mononuclear cells (PBMCs) during MeV infection shows up-regulated expression of NLRP3 and IL-1β mRNAs necessary for inflammasome activation [36]. Further in vivo evidence of the innate response during measles, are increased plasma levels of NFκB-induced proteins IL-6 and IL-8/CXCL8 [37,38] and inflammasome products IL-1β and IL-18 [37,39].

Therefore, the innate response does not include IRF-3-mediated induction of type I or III IFNs, but does include induction of a subset of NFκB- and inflammasome-associated cytokines and chemokines that are important for initiating the adaptive immune response. 

## 3. Virus Clearance

Most RNA viruses, including MeV, replicate in the cytoplasm and do not integrate their genomes into that of the host cell and are considered susceptible to immune-mediated clearance. These infections and their effects on the immune system are generally perceived to be transient and restricted to the time of virus replication, spread and clearance associated with acute disease. However, in measles viral RNA persists in lymphoid tissue and the immune system remains activated for many months (Figure 1).

Cellular immune responses are considered to be most important for clearance of MeV because children with agammaglobulinemia recover while those with defects in cellular immunity may develop progressive disease [40,41]. CD4^+^ and CD8^+^ T cell epitopes are present in many viral proteins [42,43,44] and MeV-specific cytotoxic CD8^+^ T lymphocytes, IFN-γ-producing type 1 CD4^+^ and CD8^+^ T cells, along with soluble indicators of T cell activation (e.g., β2 microglobulin, cytokines and soluble CD4, CD8 and Fas), are in circulation during the rash phase of disease when infectious virus is being cleared [9,45,46,47,48,49,50]. Depletion of CD8^+^ T cells from experimentally infected macaques results in higher and more prolonged viremias [51] and CD8^+^ T cells can control virus spread in vitro [52]. As CD4^+^ and CD8^+^ T cells infiltrate sites of virus replication [53], infectious virus decreases rapidly to undetectable levels, the rash fades and the fever resolves (Figure 1). 

However, the effector T cell response present in blood during the rash is transient, perhaps due to rapid induction of regulatory T cells as the rash is cleared, and does not result in clearance of viral RNA [9,33,42] (Figure 1). RNA persistence was first recognized during follow-up studies after hospital discharge of Zambian children with measles that discovered MeV RNA in samples from multiple sites up to four months after infectious virus was no longer recoverable [54,55]. Sequencing of RNA from late samples identified no mutations in the variable regions of either the *N* or *H* genes suggesting slow clearance as an explanation for the prolonged presence of MeV RNA after apparent recovery rather than mutational escape from the immune response [55]. 

Subsequent studies of monkeys experimentally infected with a WT strain of MeV showed persistence of MeV RNA in PBMCs for months after resolution of the rash (Figure 1) [9]. Quantitation of the MeV RNA present showed that clearance from PBMCs occurs in three to four phases (Figure 1). After an initial peak at 7–10 days (during the viremia when infectious virus can be recovered), there is a period of rapid decline coincident with clearance of infectious virus (10–14 days), followed by a rebound with up to a 10-fold increase in RNA levels (14–24 days) and then a slow decline (24–60 days) to an undetectable level. At this time LNs, and potentially other tissues, still harbour MeV RNA that can transiently reappear in PBMCs at later times [9,56,57]. 

Detailed quantitative studies of RNA clearance and the immune responses in individual macaques combined with mathematical modeling indicate that T cell responses (as indicated by IFN-γ-producing cells) correlate with clearance of infectious virus from blood, but that both antibody and T cells are required to explain the decline in viral RNA [9]. Antibody is induced to most viral proteins [58], but the relative contributions of functionally distinct antibodies and T cells for clearance from different sites are not known.

The discovery that MeV RNA persists in several locations for many weeks or months after the rash, in both children with natural measles and experimentally infected rhesus macaques, offers new insights into at least three important, but poorly understood, aspects of measles pathogenesis: prolonged immune suppression, life-long immunity and late development of progressive neurologic disease. 

## 4. Maturation of the Immune Response

**Figure 2:**
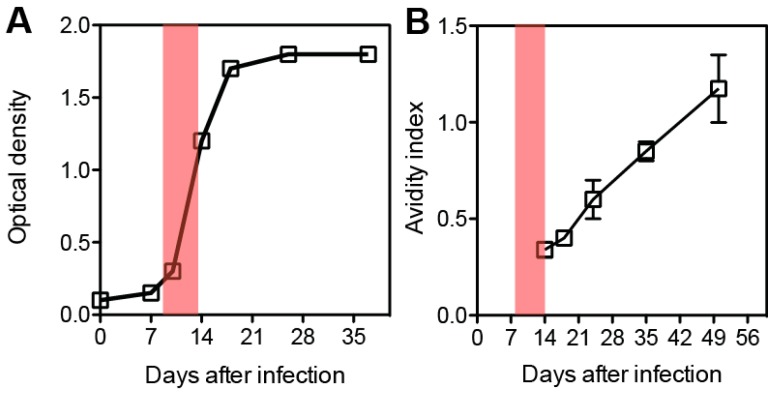
Time course for production of MeV-specific immunoglobulin G (IgG) and maturation of antibody avidity after infection. (**A**) Binding IgG antibody to MeV as determined by enzyme immunoassay; (**B**) Avidity of the antibody from panel A as measured by ammonium thiocyanate elution. Red boxes indicate the period of the rash. Data graphed from Pan et al. [65].

Continued presence of MeV RNA and proteins in lymphoid tissue after the acute phase of infection may explain suppressed immune responses to new infections, but is also likely to aid in maturation of the immune response to MeV and may be required to establish life-long protective immunity. Immune activation and lymphocyte proliferation, particularly for CD4^+^ T cells, is evident acutely and then for months after resolution of the rash [12,59]. During this period, there is a shift in cytokine production from type 1 T cell cytokines (e.g., IFN-γ) to type 2 cytokines (e.g., IL-4, IL-10, IL-13) and appearance of IL-17-producing cells [20,60,61,62] (Figure 1). This shift is likely to promote B cell maturation and contribute to the continued production of antibody-secreting cells [63]. Ongoing improvement in antibody quality, as evidenced by increasing avidity, suggests continued activity of T follicular helper (T_FH_) cells and B cell selection in the germinal centers of lymphoid tissue (Figure 2). Development of long-lived plasma cells is necessary to sustain plasma antibody levels for life [64].

## 5. Protective Immunity

Epidemiologic studies have shown that the level of neutralizing antibodies at the time of exposure to WT virus in the community is a good indicator of protection from infection with higher titers needed to prevent infection than to prevent disease (rash) [66]. High avidity antibodies are required to neutralize CD150-mediated WT MeV infection of lymphoid cells [67]. Because CD4^+^ T cell help is required for isotype and affinity maturation of antibody-secreting cells, B cell memory and maturation of CD8^+^ T cell memory, a cellular immune response is also important for the induction of protective immunity. 

T cell immunity has also been implicated as directly protective in individuals with low levels of antibody [68]. The antiviral effects of T cells can be mediated both by secretion of cytokines that suppress virus replication (e.g., IFN-γ) and by cytotoxic elimination of infected cells. Because T cells do not directly block infection but rather react to control or eliminate virus-infected cells once infection has occurred, the contribution of T cells to protection is generally considered minor in comparison to neutralizing antibodies. Furthermore, studies in macaques have shown that T cell immunity alone cannot protect from MeV infection or disease, but does facilitate RNA clearance and generation of a robust T cell and antibody response after challenge infection [69]. Therefore, generation of both immune-mediated clearance and long-lived protection requires development of effective and durable MeV-specific antibody and CD4^+^ and CD8^+^ T cell responses.
